# Joint Analysis of Multiple Traits Using "Optimal" Maximum Heritability Test

**DOI:** 10.1371/journal.pone.0150975

**Published:** 2016-03-07

**Authors:** Zhenchuan Wang, Qiuying Sha, Shuanglin Zhang

**Affiliations:** Department of Mathematical Sciences, Michigan Technological University, Houghton, Michigan, 49931, United States of America; University of North Carolina, UNITED STATES

## Abstract

The joint analysis of multiple traits has recently become popular since it can increase statistical power to detect genetic variants and there is increasing evidence showing that pleiotropy is a widespread phenomenon in complex diseases. Currently, most of existing methods use all of the traits for testing the association between multiple traits and a single variant. However, those methods for association studies may lose power in the presence of a large number of noise traits. In this paper, we propose an “optimal” maximum heritability test (MHT-O) to test the association between multiple traits and a single variant. MHT-O includes a procedure of deleting traits that have weak or no association with the variant. Using extensive simulation studies, we compare the performance of MHT-O with MHT, Trait-based Association Test uses Extended Simes procedure (TATES), SUM_SCORE and MANOVA. Our results show that, in all of the simulation scenarios, MHT-O is either the most powerful test or comparable to the most powerful test among the five tests we compared.

## Introduction

Increasing evidence shows that pleiotropy, the effect of one variant on multiple traits, is a widespread phenomenon in complex diseases [1]. Furthermore, in genetic association studies of complex diseases, multiple related traits are usually measured. For example, hyperuricemia is usually present in patients with gout [2]; coronary heart disease is predicted by cytokine interleukin-6, C-reactive protein, interleukin-1, tumor necrosis factor-α and fibrinogen [3, 4]; and neuropsychiatric disorders depend on a range of overlapping clinical characteristics [5]. Although most published genome-wide association studies (GWASs) analyze each of the related traits separately, joint analysis of multiple traits may increase statistical power to detect genetic variants [6–9]. Thus, joint analysis of multiple traits has recently become popular.

Several statistical methods have been developed for joint analysis of multiple traits. These methods can be roughly divided into three groups: combining the univariate analysis results, regression methods, and dimension reduction methods. For combining univariate analysis results, one first conducts the univariate test by performing an association test for each trait individually and then combines the univariate test statistics or combines the p-values of the univariate tests [2, 10–12]. Regression methods include mixed effect models [9, 13, 14], generalized estimating equation (GEE) methods [15, 16], and reverse regression methods [5, 17]. Mixed effect models can account for relatedness, population structure, and polygenic background effect, but it is computationally challenging. The GEE methods, based on a marginal regression model, allow the variant having different effect sizes and effect directions on different traits. These methods can also accommodate covariates and different types of traits. Reverse regression methods take genotypes as the response variable and multiple traits as independent predictors, therefore, reverse regression models do not need to know the complex distributions of traits and can be applied to a large number of mixed types of traits. Dimension reduction methods include canonical correlation analysis (CCA) [18], principal components of traits (PCT) [19], and principal components of heritability (PCH) [20–23]. CCA is to seek a linear combination of multiple variants and a linear combination of multiple traits such that the correlation between the two linear combinations reaches its maximum. The PCT methods are usually based on the first PC or first few PCs of the traits [22, 24]. However, as Aschard et al. [2014] showed that testing only the first few PCs often has low power, whereas combining signals across all PCs can have greater power. Nevertheless, it is not clear how many PCs are needed, and how robust these methods are when there exists noise traits. PCH is to find a linear combination of multiple traits such that this linear combination has the maximum heritability.

In this article, we first propose a maximum heritability test (MHT). Based on MHT, we develop an “optimal” maximum heritability test (MHT-O) to test the association between multiple traits and a single variant. In each step of MHT-O, we delete one trait that has the weakest association with the variant. Then, we find the optimal number of traits and use MHT to test the association between the optimal number of traits and the variant. Using extensive simulation studies, we compare the performance of MHT-O with MHT, Trait-based Association Test uses Extended Simes procedure (TATES) [11], SUM_SCORE and MANOVA [8]. Our results show that, in all of the simulation scenarios, MHT-O is either the most powerful test or comparable to the most powerful test among the five tests we compared.

## Method

We consider a sample with *n* unrelated individuals. Each individual has *K* (potentially correlated) traits and has been genotyped at one variant. Let *Y* = (*Y*_1_,…,*Y*_*K*_)^*T*^ denote the random vector of *K* traits and *X* denote the random variable of the genotype score at a variant. Let *y*_*i*_ = (*y*_*i*1_,…,*y*_*iK*_)^*T*^ denote the values of *K* traits and *x*_*i*_ denote the genotype score of the *i*^*th*^ individual, where *x*_*i*_ is the number of minor alleles that the *i*^*th*^ individual has at the variant. We can consider that *y*_1_,…,*y*_*n*_ is a random sample from *Y* and *x*_1_,…,*x*_*n*_ is a random sample from *X*.

Now, let us consider linear models

$$Y_{k}=\alpha_{k}+\beta_{k}X+\varepsilon_{k}\;(k=1,\ldots \;,K).$$

We partition the total phenotypic covariance of *Y* as *V*_*P*_ = *V*_*G*_ + *V*_*R*_ [25]; *V*_*G*_ = var[*β*_1_*X*,…, *β*_*K*_*X*] = var(*X*)*ββ*^*T*^ is the genetic variance due to the genotype scores *X*, where *β* = (*β*_1_,…, *β*_*K*_)^*T*^; *V*_*R*_ = var[*ε*_1_,…, *ε*_*K*_] is the residual covariance after removing the genetic effect. var(*X*) can be estimated by $\frac{1}{n}\sum_{i=1}^{n}{\left(x_{i}-\bar{x}\right)}^{2},\;\bar{x}=\frac{1}{n}\sum_{i=1}^{n}x_{i}$. *β* and *V*_*R*_ can be estimated from the linear models

$$y_{ik}=\alpha_{k}+\beta_{k}x_{i}+\varepsilon_{ik}\;(k=1,\ldots \;,K;\;i=1,\ldots \;,n).$$

*β*_*k*_ is estimated by the least square estimator. Let *r*_*ik*_ denote the estimates of residuals *ε*_*ik*_. Then, the (j, k)^*th*^ element of *V*_*R*_ is estimated by $\frac{1}{n}\sum_{i=1}^{n}r_{ij}r_{ik}$.

Let us consider a linear combination of *Y*, $w^{T}Y=\sum_{k=1}^{K}w_{k}Y_{k}$, where *w* = (*w*_1_,…,*w*_*K*_)^*T*^. The heritability of *w*^*T*^*Y* can be written as

$$h_{w}^{2}=\frac{w^{T}V_{G}w}{w^{T}V_{P}w}.$$

If we define $W=V_{P}^{1/2}w$, we can write $h_{w}^{2}$ as

$$h_{w}^{2}=\frac{W^{T}V_{P}^{-\frac{1}{2}}V_{G}V_{P}^{-\frac{1}{2}}W}{W^{T}W}=\frac{W^{T}VW}{W^{T}W},$$

where $V=V_{P}^{-\frac{1}{2}}V_{G}V_{P}^{-\frac{1}{2}}$. The heritability of *w*^*T*^*Y* depends on *w* and we can find a linear combination of *w*^*T*^*Y* that has the largest heritability among all linear combinations of *Y*. We define the maximum heritability as the test statistic to test the association between these *K* traits and the variant. We denote this test as maximum heritability test (MHT). The MHT statistic can be written as

$$T_{MHT}={\text{max}}_{w}h_{w}^{2}=\lambda_{max}\left(V_{G}V_{P}^{-1}\right)=\text{var}\left(X\right)\lambda_{max}\left(\beta \beta^{T}V_{P}^{-1}\right)=\text{var}\left(X\right)\beta^{T}V_{P}^{-1}\beta ,$$

where *λ*_*max*_(*A*) denotes the largest eigenvalue of matrix *A*.

However, the test statistic *T*_*MHT*_ may lose power in the presence of a large number of noise traits. Therefore, we propose an “optimal” maximum heritability test (MHT-O) to test the association between multiple traits and the variant. MHT-O includes a procedure of deleting traits that have weak or no association with the variant. It has the following steps:

Step 1. Given traits *Y* = (*Y*_1_,…,*Y*_*K*_), initialize *r* = *K* and *Y*^(*r*)^ = *Y*. Denote *T*_*MHT*, *r*_ as *T*_*MHT*_ based on *Y*^(*r*)^.
Step 2. Denote $T_{MHT,\;r}^{-i}$ as *T*_*MHT*_ based on *Y*^(*r*)^ with the *i*^*th*^ trait deleted for *i* = 1,…,*r*; denote $I={\text{arg max}}_{i}\;T_{MHT,\;r}^{-i}$ and $T_{MHT,\;r-1}=T_{MHT,\;r}^{-I}$. Let *Y*^(*r*−1)^ denote *Y*^(*r*)^ with the *I*^*th*^ trait deleted and update *r* = *r* − 1.
Step 3. Repeat step 2 until *r* = 1.

Denote *p*_*r*_ as the p-value of *T*_*MHT*, *r*_. The test statistic of MHT-O is defined as

$$T_{MHT-O}={\text{min}}_{1\leq r\leq K}\;p_{r}.$$

We use a permutation test to evaluate the p-value of *T*_*MHT*−*O*_. Intuitively, two layers of permutations are needed to estimate *p*_*r*_ and the overall p-value for the test statistic *T*_*MHT*−*O*_. Ge et al. [26] proposed that one layer of permutation can be used to estimate these p-values. We use the permutation procedure of Ge et al. to estimate *p*_*r*_ and the overall p-value for the test statistic *T*_*MHT*−*O*_. In details, we randomly shuffle the genotypes in each permutation. Suppose we perform *B* times of permutations. Let $T_{MHT,\;r}^{\left(b\right)}$ denote the value of *T*_*MHT*, *r*_ based on the *b*^*th*^ permuted data, where *b* = 0 represents the original data. Then, we transfer $T_{MHT,\;r}^{\left(b\right)}$ to $p_{r}^{\left(b\right)}$ by

$$p_{r}^{\left(b\right)}=\frac{\#\left\{d:T_{MHT,\;r}^{\left(d\right)}>T_{MHT,\;r}^{\left(b\right)}\;\text{for}\;d=0,1,\ldots ,B\right\}}{B}.$$

Let $p^{\left(b\right)}={\text{min}}_{1\leq r\leq K}\;p_{r}^{\left(b\right)}$, then, the p-value of *T*_*MHT*−*O*_ is given by

$$\frac{\#\{\text{b}:p^{\left(b\right)}<p^{\left(0\right)}\;\text{for}\;b=1,2,\ldots ,B\}}{B}.$$

The R code of MHT-O is available at Shuanglin Zhang’s homepage http://www.math.mtu.edu/~shuzhang/software.html.

### Comparisons of Methods

We compare our proposed method with MHT, TATES [11], MANOVA [8], and SUM_SCORE. TATES combines p-values obtained in a standard univariate GAWS to acquire one trait-based p-value, while correcting for correlations between components. SUM_SCORE performs an association test for each trait individually to obtain the univariate score test statistic for each trait. Then, the test statistic of SUM_SOCRE is the summation of the univariate score test statistics. We use asymptotic distributions to evaluate the p-values of SUM_SCORE, TATES and MANOVA.

## Simulation

To evaluate the type I error rates and powers of MHT and MHT-O, we generate genotypes according to minor allele frequency (MAF) and assume Hardy Weinberg equilibrium. Then, we generate *K* traits by the factor model [11, 19]

$$y=\lambda x+c\gamma f+\sqrt{1-c^{2}}\times \varepsilon \;,$$ (1)

where *y* = (*y*_1_,…,*y*_*K*_)^*T*^; *x* is the genotype score at the variant of interest; *λ* = (*λ*_1_,…,*λ*_*K*_) is the vector of effect sizes of the genetic variant on the *K* traits; *f* = (*f*_1_,…,*f*_*R*_)^*T*^ ∼ *MVN*(0, Σ), Σ = (1 − *ρ*)*I* + *ρA*, *A* is a matrix with elements of 1, *I* is the identity matrix, and *ρ* is the correlation between factors; **γ** is a *K* by *R* matrix; *c* is a constant number; and *ε* = (*ε*_1_,…, *ε*_*K*_)^*T*^ is a vector of residuals, and *ε*_1_,…, *ε*_*K*_ are independent, and *ε*_*k*_ ∼ *N*(0, 1) for *k* = 1,…, *K*.

Based on Eq (1), we consider five models:

Model 1: There is only one factor and genotypes impact on all traits with the same effect size. That is, *R* = 1, *λ* = (*β*,…,*β*)^*T*^, and **γ** = (1,…,1)^*T*^.

Model 2: There are five factors and genotypes impact on one factor. That is, $R=5,\;\lambda ={\left(0,\ldots ,0,\underset{K/5}{\underset{︸}{\beta ,\ldots ,\beta }}\right)}^{T}$, and **γ** = *diag*(*D*_1_, *D*_2_, *D*_3_, *D*_4_, *D*_5_), where $D_{i}={\left(\underset{K/5}{\underset{︸}{1,\ldots ,1}}\right)}^{T}$ for *i* = 1,…,5.

Model 3: There are two factors and genotypes impact on one factor. That is, $R=2,\;\lambda ={\left(0,\ldots ,0,\underset{K/2}{\underset{︸}{\beta ,\ldots ,\beta }}\right)}^{T}$, and **γ** = *diag*(*D*_1_, *D*_2_), where $D_{i}={\left(\underset{K/2}{\underset{︸}{1,\ldots ,1}}\right)}^{T}$ for *i* = 1, 2.

Model 4: There are five factors and genotypes impact on one trait. That is, *R* = 5, *λ* = (0,…,0, *β*)^*T*^, and **γ** = *diag*(*D*_1_, *D*_2_, *D*_3_, *D*_4_, *D*_5_), where $D_{i}={\left(\underset{K/5}{\underset{︸}{1,\ldots ,1}}\right)}^{T}$ for *i* = 1,…,5.

Model 5: There is only one factor and genotypes impact on one trait. That is, *R* = 1, *λ* = (0,…,0, *β*)^*T*^, and **γ** = (1,…,1)^*T*^.

To evaluate type I error rates of MHT and MHT-O, we let *β* = 0. To evaluate powers, we let *β* > 0. In the simulation studies for evaluation of type I error rates and powers, we set *MAF* = 0.3 and *ρ* = 0.2.

## Results

To evaluate the type I error rates of the two proposed tests (MHT and MHT-O), we consider 20 quantitative traits. We also consider different sample sizes, different significance levels, and different models. In each simulation scenario, the p-values of MHT and MHT-O are estimated by 1,000 permutations and the type I error rates of the two tests are evaluated using 10,000 replicated samples. For 10,000 replicated samples, the 95% confidence intervals (CIs) for estimated type I error rates of nominal levels 0.05 and 0.01 are (0.046, 0.054) and (0.008, 0.012), respectively (see Appendix for details). The estimated type I error rates of the two tests are summarized in Table 1. From this table, we can see that 58 out of 60 (greater than 95%) estimated type I error rates are within the 95% CIs and the two estimated type I error rates (0.05415 and 0.0126) not within the 95% CIs are very close to the bound of the corresponding 95% CI, which indicates that the two tests are all valid.

**Table 1:** The estimated type I error rates of MHT and MHT-O. 10,000 replicates used.

		Sample size			
			500	1000	2000
Model 1	*α* = 0.05	MHT-O	0.05415	0.0494	0.04875
		MHT	0.05235	0.05005	0.0501
	*α* = 0.01	MHT-O	0.01035	0.012	0.0091
		MHT	0.00985	0.01195	0.01105
Model 2	*α* = 0.05	MHT-O	0.0499	0.0515	0.0526
		MHT	0.04815	0.05175	0.05285
	*α* = 0.01	MHT-O	0.01045	0.01175	0.01135
		MHT	0.0117	0.0118	0.0126
Model 3	*α* = 0.05	MHT-O	0.05015	0.0517	0.05315
		MHT	0.04875	0.0507	0.0529
	*α* = 0.01	MHT-O	0.00995	0.0109	0.012
		MHT	0.0104	0.01035	0.012
Model 4	*α* = 0.05	MHT-O	0.04815	0.0516	0.05255
		MHT	0.04875	0.05275	0.0507
	*α* = 0.01	MHT-O	0.00975	0.0118	0.00975
		MHT	0.00855	0.012	0.01
Model 5	*α* = 0.05	MHT-O	0.04865	0.0499	0.04975
		MHT	0.05095	0.05195	0.04755
	*α* = 0.01	MHT-O	0.012	0.0119	0.00915
		MHT	0.01075	0.01115	0.0096

For power comparisons, we consider different values of the effect size, different models, and different numbers of traits. Sample size is 1,000 for all the cases. In each of the simulation scenarios, the p-values of MHT and MHT-O are estimated using 1,000 permutations and the p-values of SUM_SCORE, TATES and MANOVA are estimated using their asymptotic distributions. The powers of all of the five tests are evaluated using 500 replicated samples at a significance level of 0.05.

**Fig 1:**
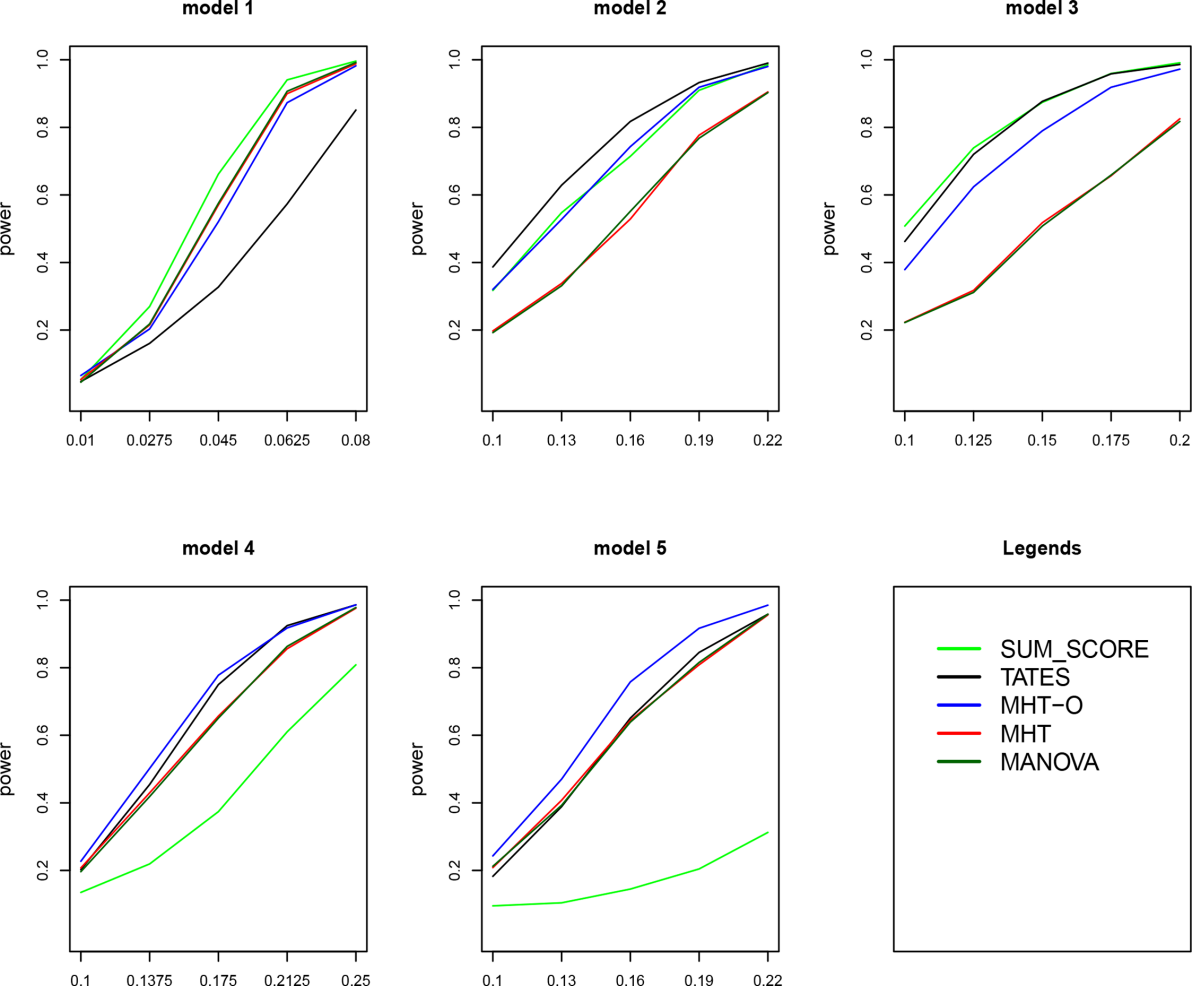
Power comparisons of the five tests (SUM_SCORE, TATES, MHT, MHT-O and MANOVA) for the power as a function of the effect size. Sample size is 1000. Total number of traits is 20.

Fig 1 gives the power comparisons of the five tests (SUM_SCORE, TATES, MHT, MHT-O and MANOVA) for the power as a function of the effect size based on the five models for 20 traits. This figure shows that (1) MHT-O is either the most powerful one (genotypes directly impact on a single trait: models 4–5) or comparable to the most powerful one (genotypes directly impact on all or a portion of the traits: models 1–3) among the five tests; (2) MHT and MANOVA have very similar powers; (3) MHT and MANOVA are much less powerful than other methods when genotypes directly impact on only a portion of the traits (models 2–3); (4) TATES is much less powerful than other methods when genotypes directly impact on all the traits (model 1); and (5) SUM_SCORE is much less powerful than other methods when genotypes directly impact on a single trait (models 4–5).

**Fig 2:**
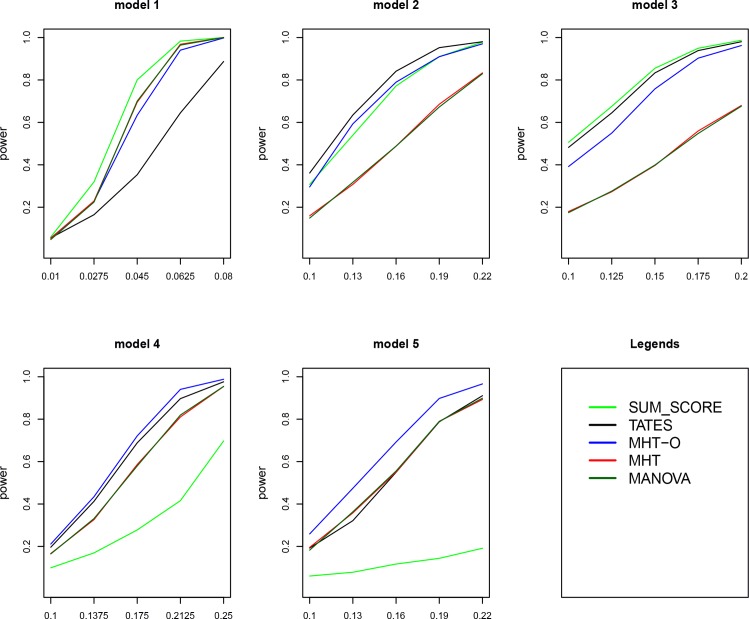
Power comparisons of the five tests (SUM_ SCORE, TATES, MHT, MHT-O and MANOVA) for the power as a function of the effect size. Sample size is 1000. Total number of traits is 30.

**Fig 3:**
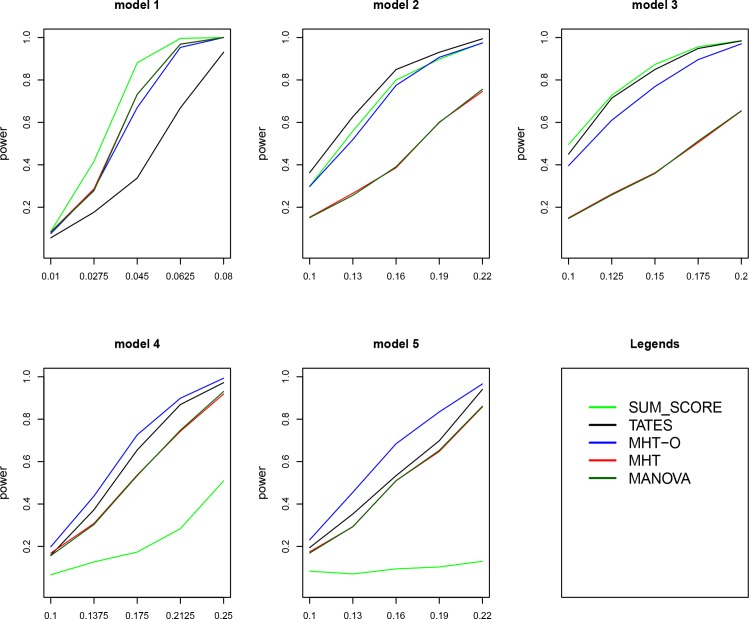
Power comparisons of the five tests (SUM_SCORE, TATES, MHT, MHT-O and MANOVA) for the power as a function of the effect size. Sample size is 1000. Total number of traits is 40.

Power comparisons of the five tests for 30 and 40 traits are given in Figs 2 and 3, respectively. The patterns of power comparisons for 30 and 40 traits (Figs 2 and 3) are similar to that for 20 traits (Fig 1). We also give power comparisons of the five tests using a significance level of 5×10^−8^ with 10^8^ permutations and 500 replicates for 20 traits under model 1 (S1 Fig). S1 Fig shows that the patterns of the power comparisons using significance level 5×10^−8^ are similar to that using a significance level of 0.05 in Fig 1 (model 1). In summary, MHT-O is either the most powerful test or comparable to the most powerful test among all the tests we compared. Therefore, our MHT-O is a robust test to a variety of models.

## Discussion

We propose MHT-O to perform joint analysis of multiple traits in association studies based on the following reasons: (1) multiple related traits are usually measured in genetic association studies of complex diseases; (2) there is increasing evidence showing that pleiotropy is a widespread phenomenon in complex diseases; and (3) the power of existing methods decreases in the presence of non-associated traits. The proposed MHT-O includes a procedure of deleting traits that have weak or no association with the variant. Therefore, it can be robust to the existence and the number of non-associated traits. By deleting one trait that has the weakest association with the variant in each step, MHT-O can maintain high power in the presence of a large number of non-associated traits. This feature is essentially important when there exist a large number of correlated traits but there are no guidelines to select relevant traits. Our results show that MHT-O has correct type I error rates and is either the most powerful test or comparable to the most powerful test among the five tests we compared. No other methods in the simulation studies show consistent good performance.

Due to the allelic heterogeneity and the extreme rarity of individual variants in rare variant association studies, the variant-by-variant methods for common variant association studies may not be optimal [27]. It has been shown by recent studies that complex diseases are caused by both common and rare variants [28–34]. Statistical methods including burden tests [27, 35–38], quadratic tests [39–41], and combined tests [42–44] have been developed for rare variant association studies with a single trait. Currently, there are limited researches on rare variant association studies for joint analysis of multiple traits [14, 45]. MHT-O can be extended to rare variant association studies by extending Eq (1) to include multiple variants. MHT-O can also be extended to family-based studies by extending Eq (1) to mixed linear model. However, the performance of MHT-O in rare variant association studies and in family-based association studies needs further investigation.

The fact that population stratification can seriously confound association results has been long recognized in association studies based on unrelated individuals [46, 47]. Several methods to control for population stratification have been developed for association studies based on unrelated individuals. These methods include principal component (PC) approach [48–52], genomic control (GC) approach [53–55], and mixed linear model (MLM) approach [29, 56]. Like most association tests based on unrelated individuals, MHT-O subjects to bias due to population stratification. To make MHT-O robust to population stratification, we can use the PC approach. Let *P*_*i*_ = (*p*_*i*1_,…,*p*_*iL*_)^*T*^ denote the first *L* PCs of the genotypes at a set of genomic markers for the *i*^*th*^ individual. Let $y_{ik}^{*}$ and $x_{i}^{*}$ denote the residuals of the regressions $y_{ik}=\alpha_{0k}+\alpha_{k}^{T}P_{i}+\varepsilon_{ik}$ and the residuals of the regression *x*_*i*_ = *α*_0_ + *α*^*T*^*P*_*i*_ + *ε*_*i*_, respectively. Using $y_{ik}^{*}$ and $x_{i}^{*}$ to replace *y*_*ik*_ and *x*_*i*_, we can make MHT-O robust to population stratification. However, the performance of using the PC approach to control for population stratification in MHT-O needs further investigations.

## Appendix

Let *p* denote the p-value of the test and denote a random variable

$$\xi =\{\begin{array}{ll} 1, & p\leq \alpha \\ 0, & p>\alpha \end{array}\;,$$

where *α* is the significance level. Then, Pr(*ξ* = 1) = *α* and Pr(*ξ* = 0) = 1 − *α* because *p* follows a uniform distribution between 0 and 1 under the null hypothesis. Suppose there are *R* replicates. Let *ξ*_*i*_ denote the value of *ξ* for the *i*^*th*^ replicate, where *i* = 1,…,*R* Then, the estimated type I error rate is given by $\bar{\xi }=\frac{1}{R}\sum_{i=1}^{R}\xi_{i}$ that asymptotically follows a normal distribution $N\left(\alpha ,\frac{\alpha \left(1-\alpha \right)}{R}\right)$. Thus, $\text{Pr}\left(\left|\frac{\bar{\xi }-\alpha }{\sqrt{\alpha \left(1-\alpha \right)/R}}\right|\leq 1.96\right)=\text{Pr}\left(\alpha -1.96\sqrt{\alpha \left(1-\alpha \right)/R}\leq \bar{\xi }\leq \alpha +1.96\sqrt{\alpha \left(1-\alpha \right)/R}\right)=0.95$.

We define $\left(\alpha -1.96\sqrt{\alpha \left(1-\alpha \right)/R},\alpha +1.96\sqrt{\alpha \left(1-\alpha \right)/R}\right)$ as the 95% confidence interval for the estimated type I error rate for the nominal level *α*.

## Supporting Information

S1 Fig Power comparisons of the five tests (SUM_SCORE, TATES, MHT, MHT-O and MANOVA) for the power as a function of the effect size (model 1). Sample size is 1000. Total number of traits is 20. The significance level is 5×10^−8^. The number of replicates is 500. The number of permutations is 10^8^.
(EPS)
